# Invasive ductal carcinoma of the breast presenting as a cauliflower-like growth

**DOI:** 10.11604/pamj.2024.48.4.43431

**Published:** 2024-05-02

**Authors:** Keval Dhone, Gaurang Aurangabadkar

**Affiliations:** 1 Department of General Surgery, Datta Meghe Medical College, Nagpur, Datta Meghe Institute of Higher Education and Research (DMIHER), Deemed University (DU), Wardha, Maharashtra, India,; 2 Department of Respiratory Medicine, Datta Meghe Medical College, Nagpur, Datta Meghe Institute of Higher Education and Research (DMIHER), Deemed University (DU), Wardha, Maharashtra, India

**Keywords:** Invasive ductal carcinoma, excisional biopsy, neoadjuvant chemotherapy, cauliflower-like growth

## Image in medicine

A female patient aged 50 years presented to the general surgery outpatient department (OPD) with a history of progressively increasing growth involving the right breast. The patient had first noticed this growth 8 months back, and was progressively increasing in size as per history given by the patient. On breast examination, a cauliflower-like growth was visible on the surface of the right breast, involving the chest wall and nipple as well. For establishing tissue diagnosis, an excisional biopsy was taken from the lesion and sent for histopathological examination, which established the diagnosis of invasive ductal carcinoma (IDC) of the breast. The tumor-node-metastasis (TNM) staging of the breast cancer was found to be stage 3B and an oncologist’s opinion was sought promptly, and the patient was advised neo-adjuvant chemotherapy with regular follow-up. Surgical excision could not be attempted as the tumor was found to be fixed to the chest wall, and in a locally advanced stage. Invasive ductal carcinoma has been found to be one of the commonest variants of carcinoma of the breast. This patient had a rare presentation with a cauliflower-like growth involving the right breast and the chest wall.

**Figure 1:**
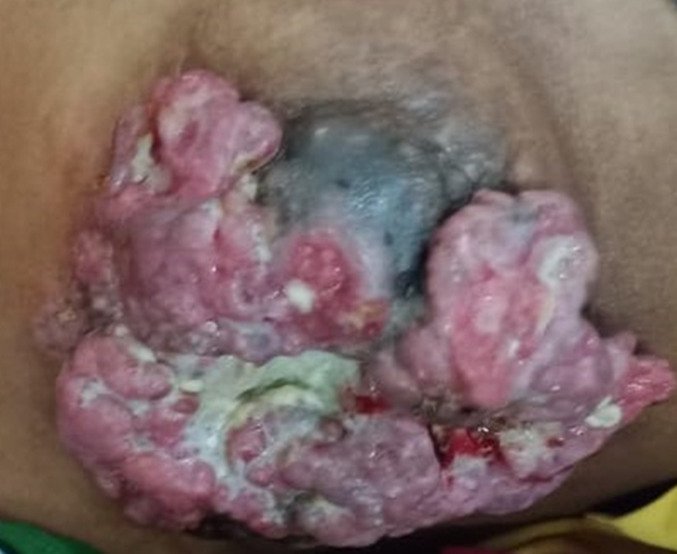
gross appearance of the right breast showing the presence of a cauliflower-like growth with involvement of the nipple and the chest wall

